# Significant Phylogenetic Signal and Climate-Related Trends in Leaf Caloric Value from Tropical to Cold-Temperate Forests

**DOI:** 10.1038/srep36674

**Published:** 2016-11-18

**Authors:** Guangyan Song, Ying Li, Jiahui Zhang, Meiling Li, Jihua Hou, Nianpeng He

**Affiliations:** 1 The Key Laboratory for Forest Resources & Ecosystem Processes of Beijing, Beijing Forestry University, Beijing 100083, China; 2 Key Laboratory of Ecosystem Network Observation and Modeling, Institute of Geographic Sciences and Natural Resources Research, Chinese Academy of Sciences, Beijing 100101, China

## Abstract

Leaf caloric value (LCV) is a useful index to represent the conversion efficiency of leaves for solar energy. We investigated the spatial pattern of LCV and explored the factors (phylogeny, climate, and soil) that influence them at a large scale by determining LCV standardized by leaf area in 920 plant species from nine forest communities along the 3700 km North-South Transect of Eastern China. LCV ranged from 0.024 to 1.056 kJ cm^−2^ with an average of 0.151 kJ cm^−2^. LCV declined linearly with increasing latitude along the transect. Altogether, 57.29% of the total variation in LCV was explained by phylogenetic group (44.03% of variation), climate (1.27%), soil (0.02%) and their interacting effects. Significant phylogenetic signals in LCV were observed not only within forest communities but also across the whole transect. This phylogenetic signal was higher at higher latitudes, reflecting latitudinal change in the species composition of forest communities from complex to simple. We inferred that climate influences the spatial pattern of LCV through directly regulating the species composition of plant communities, since most plant species might tolerate only a limited temperature range. Our findings provide new insights into the adaptive mechanisms in plant traits in future studies.

Caloric value is a measure of energy content, and the caloric content of leaves (leaf caloric value, LCV) might thus reflect the converting efficiency of plants to solar energy through photosynthesis to some extent^1^. Studies of LCV began in the 1930s^2^ and have since been conducted on species typical of grasslands, forests, mangroves, and other habitats^3^,^4^,^5^. However, thus far, most studies focused on the pattern of LCV at a small scale, and the few that explore larger-scale trends have been limited to two or three study sites. Some larger-scale studies have found that LCV increases with increasing latitude in some plant species^1^,^6^,^7^, but these results are inconsistent with the findings of recent studies^8^. These issues can be addressed by conducting, a systematic investigation of LCV at a large spatial scale.

Although large-scale patterns remain ambiguous, several studies have explored the mechanisms that might underpin smaller-scale variation in LCV. For example, LCV was correlated closely with leaf element content (i.e., carbon and nitrogen^9^,^10^). Environmental factors (soil and nutrition availability) might further influence LCV to some extent^5^,^11^. However, such studies were mainly focused on the effect of single factor on a few plant species or a single plant community, with few previous studies considering the influence of multiple factors and at a large scale.

In addition to extrinsic influences on LCV, some recent studies have shown that plant traits are influenced by phylogeny^12^,^13^. This means that the influence of phylogenetic history needs to be considered for the analyses of plant traits, because trait stability in a plant community results from the long-term evolution and adaptation of each plant species to their biotic and abiotic environments. Although LCV is considered a relatively stable plant trait, it differs across species^3^,^14^. Moreover, the strength of phylogenetic signals in plant traits is increasingly used to characterize the genetic relationships between plant species in a community. However, the species composition of a plant community is shaped by environmental filtering as well as phylogenetic history^15^. For example, climate can regulate plant species composition because most plant species can only tolerate a limited temperature range^16^,^17^. The difference in soil condition might also affect the species composition of a community at a large scale. Large-scale spatial patterns in LCV may therefore result from both the large-scale differences in the species composition of plant communities and the between-species variations in LCV. To date, the relative contributions of phylogeny, climate, and soil to the spatial patterns in LCV remain unclear.

In this study, we investigated the variations in LCV across 920 plant species from nine typical forest communities along the North-South Transect of Eastern China (NSTEC), covering forests from tropical to cold-temperate biomes across 33° of latitude (Table 1 and Fig. S1). This study mainly aimed to (1) reveal the spatial patterns in LCV at a large scale; (2) explore the main factors influencing LCV (phylogeny, climate, and soil); and (3) test the hypothesis that climate can indirectly affect large-scale spatial variation in LCV via its influence on the species composition of plant communities.

## Results

**Figure 1:**
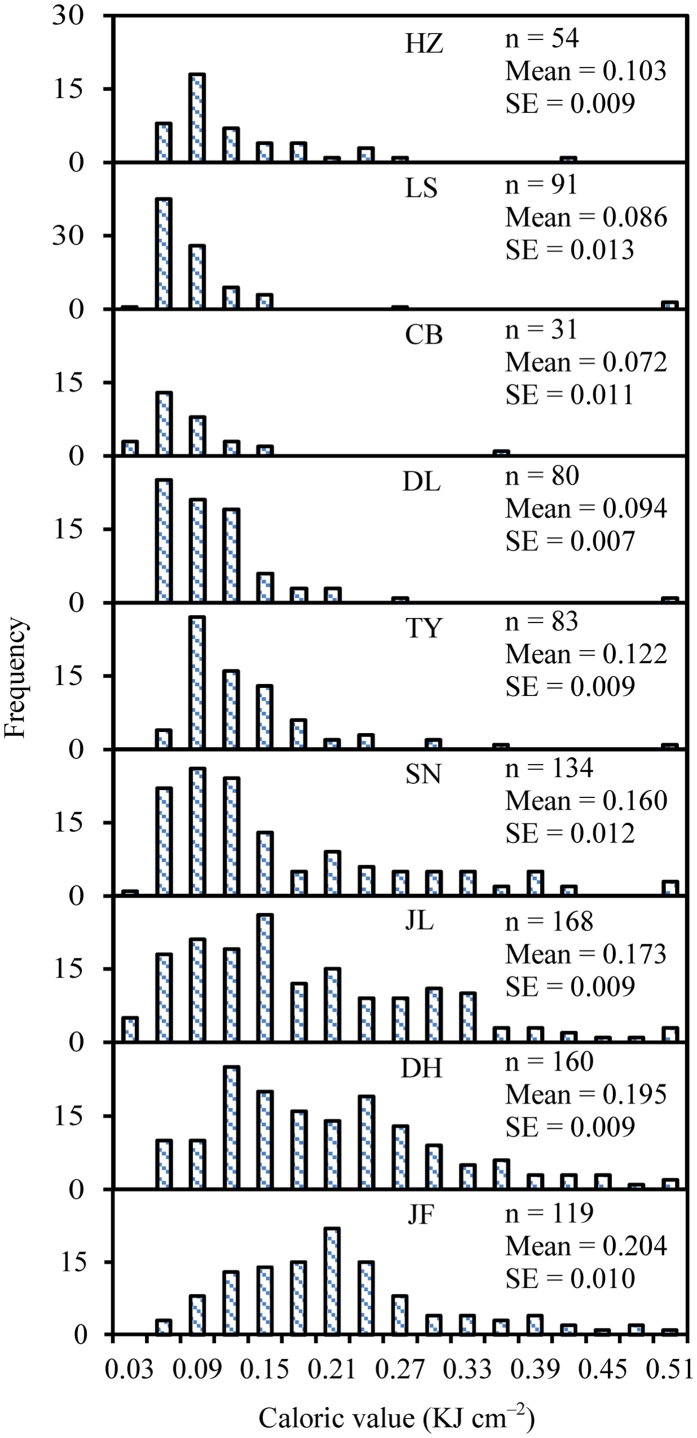
Frequency distribution of species-specific leaf caloric value in the nine forest communities along the transect. *n* is the number of the species, SE is the standard error. HZ, Huzhong; LS, Liangshui; CB, Changbai; DL, Dongling; TY, Taiyue; SN, Shennongjia; JL, Jiulian; DH, Dinghu; JF, Jianfengling.

**Figure 2:**
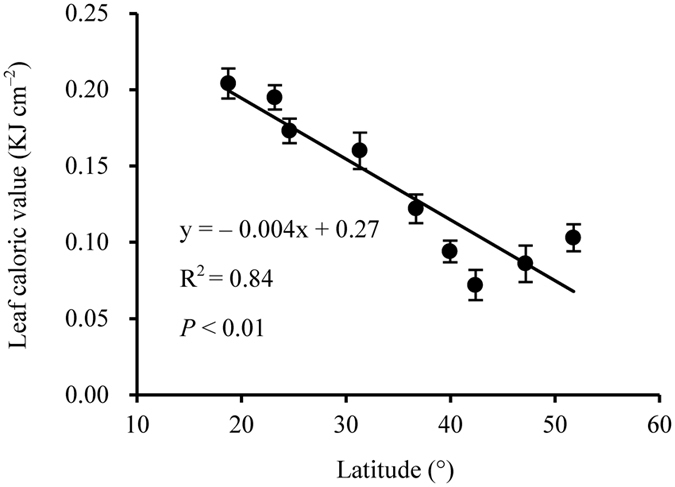
The relationship of leaf caloric value with latitude along the forest transect (mean ± standard error).

Across the 920 species, LCV ranged from 0.024 to 1.056 kJ cm^−2^ with an average of 0.151 kJ cm^−2^ (Figs S2 and S3). Plant species with high LCV decreased owing to frequent distribution from tropical monsoon forest to cold-temperate coniferous forest (Fig. 1), yielding a significant decrease in LCV with increasing latitude (R^2^ = 0.84, *P* < 0.01; Fig. 2).

LCV varied markedly and significantly across phylogenetic groups, i.e., gymnosperms (high LCV, 0.470 ± 0.03 kJ cm^−2^), angiosperms (medium LCV, 0.144 ± 0.048 kJ cm^−2^), and ferns (low LCV, 0.102 ± 0.010 kJ cm^−2^; *P* < 0.05, Table 2).

**Figure 3:**
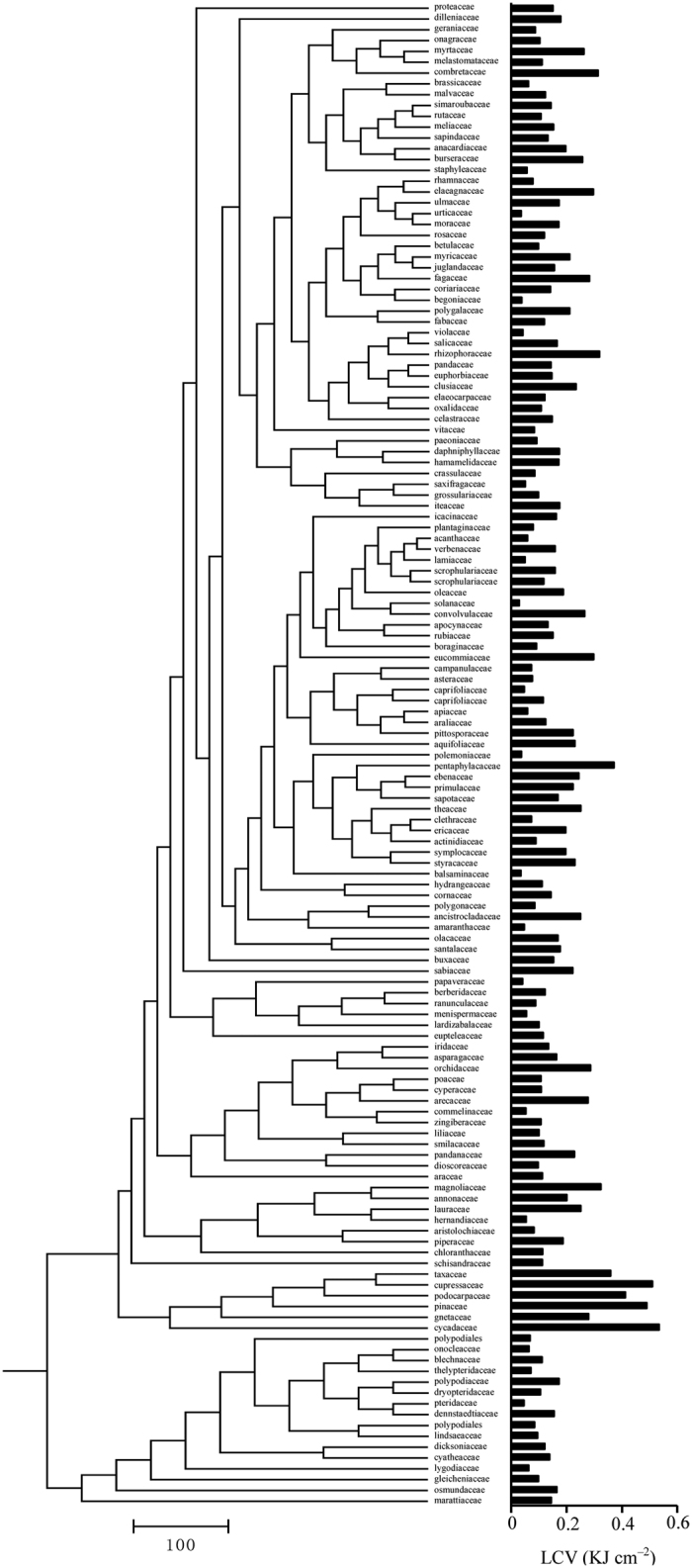
Phylogenetic tree of the 139 plant families sampled.

At the family level, the gymnosperms Cycadaceases (0.534 kJ cm^−2^), Cupressaceae (0.509 kJ cm^−2^), Pinaceae (0.489 kJ cm^−2^), and Podocarpaceae (0.411 kJ cm^−2^) had relative high LCV. Among the angiosperms families, the ancient Magnoliaceae (0.323 kJ cm^−2^) had high LCV, whereas Solanaceae (0.028 kJ cm^−2^), Balsaminaceae (0.034 kJ cm^−2^), and Urticaceae (0.036 kJ cm^−2^) had low LCV. Among ferns, the family Marattiaceae (0.144 kJ cm^−2^) had high LCV and Pteridaceae (0.045 kJ cm^−2^) had low LCV (Fig. 3).

At the community level, LCV showed significant phylogenetic signals not only for each forest type but also across the entire forest belt (all *P* < 0.05; Table 3). Furthermore, the strength of the phylogenetic signal in LCV decreased linearly with increasing latitude (R^2^ = 0.91, *P* < 0.01; Fig. S4).

**Figure 4:**
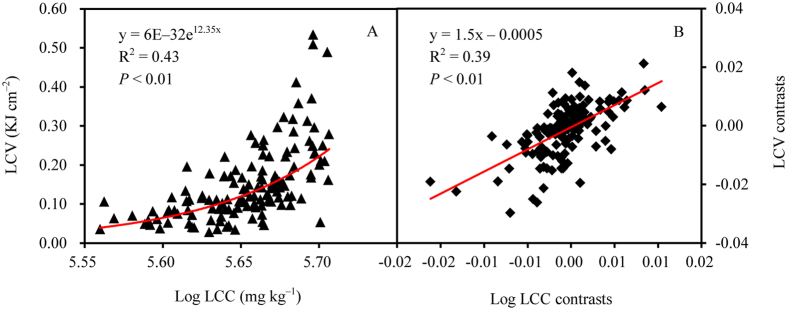
The relationships between leaf caloric value (LCV) and leaf carbon content (LCC) determined using a simple regression (**A**) and excluding phylogenetic effects by using phylogenetically independent contrasts (**B**).

**Figure 5:**
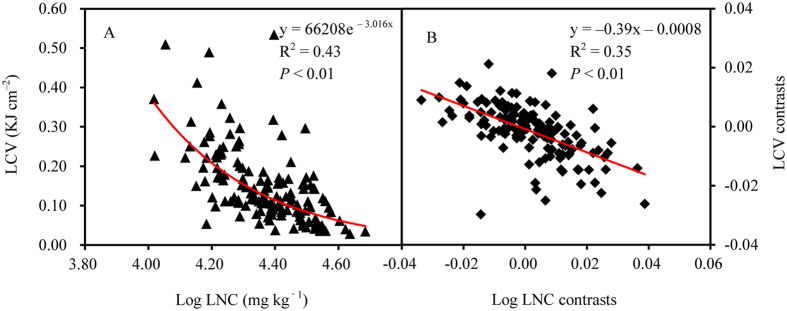
The relationships between leaf caloric value (LCV) and leaf nitrogen content (LNC) determined using a simple regression (**A**) and excluding phylogenetic effects by using phylogenetically independent contrasts (**B**).

LCV was significantly correlated with leaf element content (leaf carbon content, LCC; leaf nitrogen content, LNC), climatic factors (mean annual temperature, MAT; mean annual precipitation, MAP), and soil factors (soil total carbon, STC; soil total nitrogen, STN; Table S1). Detailed examination showed that LCV significantly increased exponentially with LCC but declined exponentially with increasing LNC (Figs 4A and 5A). When phylogenetic effects were removed in the PIC analysis, LCV and LCC showed a positive linear relationship, whereas LCV and LNC showed a negative linear relationship (all *P* < 0.01; Figs 4B and 5B).

With relation to climatic variables, LCV only increased significantly with increasing MAT and MAP in fewer than half of the examined families (Fig. S5, Table S2), although the average LCV in each forest type was significantly positively correlated with both the factors (*P* < 0.01; Fig. S6). Furthermore, the average LCV had significant negative correlations with soil element content (STC and STN) along the forest transect (*P* < 0.01; Fig. S7). No relationship was found between LCV and soil pH.

**Figure 6:**
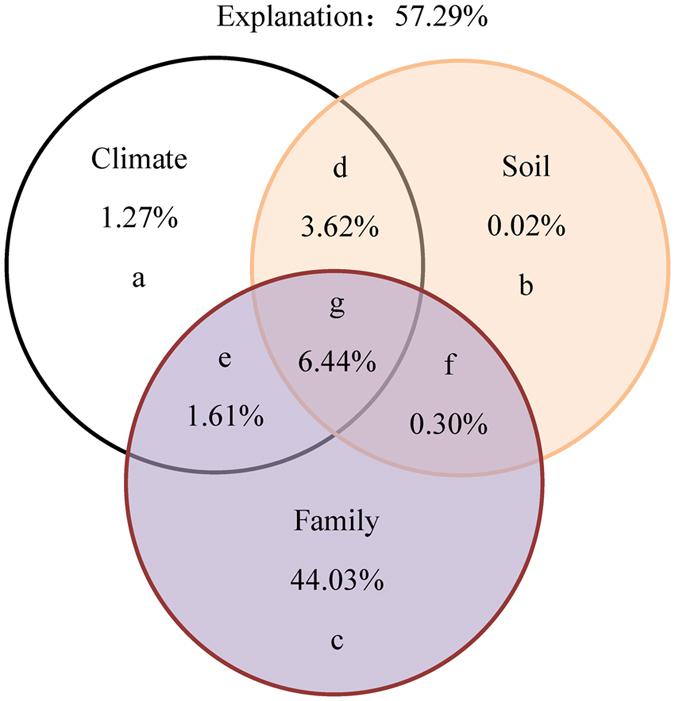
The contributions of phylogenetic group, climate, and soil to variations in leaf caloric value (LCV). Areas (**a**–**c)** show single effects and (**d**–**g)** show interaction effects.

Across the forest belt, 57.29% of the total variation in LCV was explained by the simultaneous effects of phylogenetic group, climate, and soil factors (Fig. 6). In particular, phylogenetic group, climate, and soil element content might explain 44.03%, 1.27%, and 0.02% of the total variation, respectively. In addition, the combined effects of climate and phylogenetic group explained 46.91% of the variation in LCV; the those of climate and soil 4.91%; and the those of soil element content and phylogenetic group 44.35%.

## Discussion

In this study, we conducted a uniquely broad exploration of variation in LCV at a range of spatial scales and phylogenetic levels. We found that, from tropical monsoon forest to cold-temperate coniferous forest, species-specific LCV decreased with increasing latitude (Figs 1 and 2). This trend was accompanied by a latitudinal decline in the number of plant species in each community. A similar decrease in LCV with increasing latitude was described in a single-species study of the LCV of *Kandelia candel* at eight sites^18^. However, previous studies comparing tropical monsoon forest, temperate forests, and alpine vegetation found that LCV increased with increasing latitude^1^,^6^. In contrast to these ambiguous studies, which only investigated a few species in each community, our objectives were to cover a wide phylogenetic range (Fig. 1). The resulting difference between studies in the number of sampled species might explain the different patterns of LCV observed. Moreover, previous research in this field focused on high latitudes and even polar regions^19^, with sampling often conducted after autumn, when low temperatures would promote high accumulation of caloric substances in leaves. Our comprehensive and systematic study remarkably contributes to resolving these inconsistencies.

At different phylogenetic levels, LCV had a significant phylogenetic signal (Table 3). Across plant functional ecology, a growing body of evidence shows that phylogeny can influence plant traits (e.g., flowering phenology and nutrient contents)^20^,^21^,^22^ as well as correlations between plant traits^13^,^23^. Indeed, when we excluded phylogenetic effects, the correlation of LCV with LCC and LNC changed from exponential to linear (Figs 4 and 5), indicating an influence of phylogeny on the correlation between LCV and leaf element content. Overall, a significant relationship between LCV and LCC is consistent with the findings of previous studies^9^,^24^. Further, as proposed by Han *et al*.^25^, we found that LNC increased with increasing latitude, which is consistent with our observation that LCV linearly decreased with increasing latitude. However, unlike leaf ash content, LCV was not closely correlated with soil pH^11^.

Interestingly the phylogenetic signal of LCV in each forest community became higher further north. Du *et al*.^20^ investigated variation in the phylogenetic signal in flowering phenology and similarly found that the phylogenetic signal tended to be higher towards the temperate regions. These observed latitudinal patterns could be explained by the phylogenetic niche conservatism hypothesis, which posits that plant species tend to be more phylogenetically clustered and individuals tend to be younger in colder regions^26^. Such phylogenetic clustering would render LCV to be more similar in communities at higher latitudes composed of more closely related species. Moreover, there is a general consensus that species richness is higher at lower latitudes^27^, which was also supported by the distribution of LCV in this study (Fig. 1). LCV also differed among species, again with a significant phylogenetic signal. Thus, the observed spatial pattern in LCV through the forest belt is well explained by the fact that lower latitudes harbor a greater number of plant species, each with higher LCV.

When considering latitudinal patterns in species richness, we also need to consider that in natural ecosystems, the species composition of plant communities is mainly controlled by large-scale climatic and soil factors^28^,^29^. Any given plant species is known to has a limited range of temperature tolerance, which limits species distributions^16^. In this study, most plant species and even families were only present in two or four forest communities along the transect (Fig. S5). This suggests that climate indirectly influenced the spatial variation in LCV by regulating species composition of communities at a large scale. However, despite this putative importance of climate in explaining LCV patterns, we found that phylogenetic group explained most variation in LCV, with climate explaining very little (Fig. 6). Previous studies have shown that soil carbon and contents were also affected by climate along the transect^30^, which was consistent with our results (Table S1). Therefore, we assumed that climate had remarkable indirect effects on LCV by altering soil factors and species composition of communities in the forest transect. Further studies are warranted to further verify this assumption by investigating similar trends in other plant traits, such as leaf morphological and stomatal traits, at a similarly large scale.

## Conclusion

LCV declined from tropical monsoon forest to cold-temperate coniferous forest, although it varied significantly between different plant species. Our phylogenetic analysis independently confirmed that LCV is mainly constrained by phylogeny. Furthermore, phylogenetic conservatism of forest LCV was more marked in temperate regions than in tropical regions. At this large scale, climate indirectly affected the spatial patterns of LCV in forest communities because of its important influence on species composition. Our findings provide new insights that might form the basis for future explorations of the adaptive mechanisms of plants traits.

## Materials and Methods

### Site description

The North-South Transect of Eastern China (NSTEC) is a unique forest belt exhibiting a cline in biomes from tropical monsoon forest to cold-temperate coniferous forest, shaped mainly by a thermal gradient. In this study, nine typical natural forests along a 3700 km stretch of the NSTEC were sampled (Fig. S1): Huzhong (HZ, cold temperate coniferous forest), Liangshui (LS, temperate conifer broad-leaved mixed forest), Changbai (CB, temperate conifer broad-leaved mixed forest), Dongling (DL, warm temperate deciduous broad-leaved forest), Taiyue (TY, warm temperate deciduous broad-leaved forest), Shennongjia (SN, subtropical deciduous evergreen mixed forest), Jiulian (JL, subtropical evergreen broad-leaved forest), Dinghu (DH, subtropical monsoon evergreen broad-leaved forest), and Jianfengling (JF, tropical monsoon forest). These forests span latitudes 18.7–51.8 °N and longitudes 108.8–123.0 °E. The mean annual temperature (MAT) along the transect ranged from −4.4 °C to 20.9 °C, and the mean annual precipitation (MAP) ranged from 481.6 to 2449.0 mm. Soil properties of each site are shown in Table 1.

### Field sampling

Field surveys were conducted between July and August 2013. We collected leaf samples from nine forest communities according to a standard protocol^31^. At each site, we defined four 30 m × 40 m plots in which plants were sampled. We tried to collect all species present in the plots. For herbaceous plants, the whole plant was collected. For woody plants, we chose healthy mature trees climbed or used high branch shears to collect canopy leaves from four different directions. The leaves of each plant species were pooled for each plot^32^. In total, we collected samples from 920 plant species from nine forest communities, which consisted of 745 species in 139 families (replicated species not considered). Soil samples (0–10 cm depth) were randomly collected from 30–50 points by using a soil sampler (diameter 6 cm) in each plot and combined to form one composite sample per plot^33^,^34^.

### Measurements

Leaf samples were cleaned to remove soil and other contaminants. We randomly selected ten fresh leaves for each plant species, scanned them using a scanner (CanoScan LiDE 110, Canon, Japan) and measured their area by using Photoshop CS (Adobe Systems, San Jose, USA). For these compound leaves, they were scanned as an integral; if the compound leaves were very large for the scanner, they were divided into several leaflets to scan and then summed up. The leaves were then oven-dried at 60 °C until they reached a constant weight and weighed to calculate the specific leaf area (SLA, mm^2^ mg^−1^)^35^.

LCV per leaf mass (LCV_*mass*_) of samples was measured using a Parr 6300 automatic isoperibol calorimeter (Parr Instrument Company, Moline, IL, USA). The carbon and nitrogen content of leaf (LCC, LNC) and soil samples (STC, STN) were measured using an elemental analyzer (Vario MAX CN Elemental Analyzer, Elementar, Germany)^36^. Soil pH was determined using a pH meter (Mettler Toledo Delta 320, Switzerland) by using a slurry of soil and distilled water (1:2.5).

In order to better reflect the capacity of leaves to capture solar radiation, we calculated leaf caloric value per area (LCV_*area*_, kJ cm^−2^) based on SLA (Equation 1):


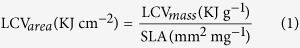


where LCV_*mass*_ is LCV per mass and LCV_*area*_ is LCV per area. Henceforth, we use LCV to mean LCV_*area*_.

The climatic variables (MAT and MAP) were extracted from the data from 740 climate stations of the China Meteorological Administration during 1961 and 2007 using the interpolation software ANUSPLIN^37^.

### Phylogeny

We constructed a phylogenetic tree at the species and family levels by using the data from 745 species. By using the Latin name of each species as given in the Plant List (http://www.theplantlist.org/), we determined the order, family, and genus of each species based on the Angiosperm Phylogeny Group III classification (APG III)^38^. We defined a reference phylogenetic tree and resolved it to family and species level by using the freely available software Phylomatic v3 (http://phylodiversity.net/phylomatic/)^39^. Branch lengths were determined using the Branch Length Adjuster algorithm in Phylocom^40^.

### Statistical analysis

Differences in LCV between different phylogenetic groups were tested using one-way analysis of variance with a test for least significant difference. The strength of the phylogenetic signal in LCV across the sample was quantified using Blomberg’s *K* statistic^41^ which tests whether the observed trait variation across a phylogeny is smaller than expected according to a Brownian motion model of trait evolution. We tested the significance of this phylogenetic signal by comparing the actual system to a null model without a phylogenetic structure. If the real value of the phylogenetic signal in the trait was greater than 95% of that of the null model (*P* < 0.05), the phylogenetic signal was considered significant, and vice versa. The phylogenetic signal was quantified and tested using the ‘picante’ package in R^42^.

Regression analyses were conducted to test for a latitudinal pattern in LCV and for a phylogenetic signal at the community level. Relationships between LCV and influencing factors were assessed using Pearson correlations. The relationship between LCV and leaf element content was tested using phylogenetically independent contrasts (PIC) after the phylogenetic effect was excluded^43^. PIC correlation coefficients were calculated using the ‘pic’ package in the R. The relationship between LCV and climate used only those families that appeared in at least three forest types. The relationships between LCV at plot level and climate and soil factors were explored using linear regressions.

The effect of climate (MAT and MAP), soil (STC and STN), and phylogeny (family level) on the spatial variation of LCV were further quantified using general linear models (GLMs) and partial GLMs. To avoid collinearity among explanatory variables, we removed correlated predictors by using multiple stepwise regressions (*P* < 0.05). Partial GLMs were then used to divide the explanatory power of these factors into independent and interactive effects^44^.

All tests used a significance level of *P* = 0.05. All analyses were conducted using the software SPSS 13.0 (SPSS Inc., Chicago, IL, USA, 2004) or R (version 2.15.2, R Development Core Team 2012). All figures were produced in SigmaPlot 10.0 (Washington, IL, USA, 2006).

## Additional Information

**How to cite this article**: Song, G. *et al*. Significant Phylogenetic Signal and Climate-Related Trends in Leaf Caloric Value from Tropical to Cold-Temperate Forests. *Sci. Rep.* **6**, 36674; doi: 10.1038/srep36674 (2016).

**Publisher’s note:** Springer Nature remains neutral with regard to jurisdictional claims in published maps and institutional affiliations.

## Supplementary Material

Supplementary Information

## Figures and Tables

**Table 1:** Characteristics of the nine forests along the North-South Transect of Eastern China.

Site	Latitude (°N)	Longitude (°E)	MAT† (°C)	MAP (mm)	Vegetation type	Soil type
JF‡	18.74	108.86	19.8	2449.0	Tropical monsoon forest	Laterite soil
DH	23.17	112.54	20.9	1927.1	Subtropical monsoon evergreen broad- leaved forest	Lateritic red soil
JL	24.58	114.44	16.7	1954.2	Subtropical evergreen broad-leaved forest	Red soil
SN	31.32	110.50	10.6	1330.0	Subtropical deciduous evergreen mixed forest	Yellow-brown soil
TY	36.70	112.08	6.2	662.6	Warm temperate deciduous broad-leaved forest	Cinnamon soil
DL	39.96	115.42	4.8	539.1	Warm temperate deciduous broad-leaved forest	Brown soil
CB	42.40	128.09	2.6	690.9	Temperate conifer broad-leaved mixed forest	Dark brown soil
LS	47.19	128.90	−0.3	675.8	Temperate conifer broad-leaved mixed forest	Dark brown soil
HZ	51.78	123.02	−4.4	481.6	Cold temperate coniferous forest	Grey forest soil

^†^MAT: mean annual temperature; MAP: mean annual precipitation.

^‡^HZ, Huzhong; LS, Liangshui; CB, Changbai; DL, Dongling; TY, Taiyue; SN, Shennongjia; JL, Jiulian; DH, Dinghu; JF, Jianfengling.

**Table 2:** Leaf caloric value (LCV) in the sampled phylogenetic groups.

	n†	LCV (KJ cm^−2^)	Min	Max	CV
Phylogeny					
Pteridophyta	45	0.102 ± 0.010^a,,^‡	0.026	0.292	0.064
Gymnosperm	26	0.470 ± 0.003^b^	0.126	1.056	0.052
Angiosperm	849	0.144 ± 0.048^c^	0.024	0.621	0.066
Angiosperm Phylogeny Group§					
Angiosperm	6	0.118 ± 0.022	0.032	0.190	0.046
Magnoliids	50	0.246 ± 0.013	0.053	0.426	0.038
Monocots	45	0.112 ± 0.010	0.037	0.318	0.059
Commelinids	48	0.118 ± 0.011	0.037	0.459	0.067
Eudicots	41	0.103 ± 0.012	0.024	0.401	0.077
Coreeudicots	26	0.119 ± 0.013	0.034	0.249	0.055
Rosids	10	0.082 ± 0.018	0.042	0.235	0.068
Fabids	279	0.147 ± 0.006	0.025	0.481	0.063
Malvids	77	0.158 ± 0.010	0.038	0.464	0.058
Asterids	89	0.186 ± 0.011	0.034	0.465	0.054
Lamiids	72	0.138 ± 0.013	0.027	0.621	0.078
Campanulids	108	0.103 ± 0.007	0.031	0.407	0.074

^†^n, number of plant species; CV, coefficient of variation.

^‡^Data represented as mean ± SD, standard deviation and different letters indicate significant difference in phylogeny groups at *P *= 0.05 level.

^§^The angiosperm phylogeny groups were divided as APG III (Group 2009).

**Table 3:** Strength of the phylogenetic signal in LCV for each of the nine forest communities.

	n†	*K-*value	*P*
HZ‡	54	1.11	<0.01
LS	91	1.27	<0.01
CB	31	0.85	0.02
DL	80	0.82	<0.01
TY	83	0.67	0.02
SN	134	0.65	<0.01
JL	168	0.30	<0.01
DH	160	0.28	0.03
JF	119	0.33	0.02
Total	754	0.37	<0.01

^†^n is the number of the species, *K-*value is phylogenetic signal, *P* is the significance level;

^‡^HZ, Huzhong; LS, Liangshui; CB, Changbai; DL, Dongling; TY, Taiyue; SN, Shennongjia; JL, Jiulian; DH, Dinghu; JF, Jianfengling; Total is the whole transect.
