# Supplementary material for: Significant Phylogenetic Signal and Climate-Related Trends in Leaf Caloric Value from Tropical to Cold-Temperate Forests
Source: Sci Rep. 2016 Nov 18;6:36674. doi: 10.1038/srep36674 (PMC5114554; doi:10.1038/srep36674)
Supplement: Supplementary Information [file srep36674-s1.pdf]

Significant Phylogenetic Signal and Climate-Related Trends in Leaf Caloric Value from Tropical to Cold-Temperate Forests

Guangyan Song[1, 2], Ying Li[1], Jiahui Zhang[2], Meiling Li[2], Jihua Hou [1*], Nianpeng He [2*]

1 The Key Laboratory for Forest Resources & Ecosystem Processes of Beijing, Beijing Forestry University, Beijing 100083, China

2 Key Laboratory of Ecosystem Network Observation and Modeling, Institute of Geographic Sciences and Natural Resources Research, Chinese Academy of Sciences, Beijing 100101, China

**Corresponding author*. Nianpeng He and Jihua Hou

Institute of Geographic Sciences and Natural Resources Research, Chinese Academy of Sciences,

Beijing 100101, China

Tel.: +86 10 64889263

Fax: +86 10 68489432

E-mail address: henp@igsnrr.ac.cn; houjihua@bjfu.edu.cn

Table S1: Pearson's coefficients between leaf caloric value and main influencing factors

	LCV	LCC	LNC	MAT	MAP	STC	STN	pH
Leaf caloric value (LCV)	1.00							
Leaf carbon content (LCC)	0.78*	1.00						
Leaf nitrogen content (LNC)	-0.85**	–0.69**	1.00					
Mean annual temperature (MAT)	0.90**	0.64	–0.72*	1.00				
Mean annual precipitation (MAP)	0.93**	0.78*	–0.81**	0.93**	1.00			
Soil total carbon (STC)	-0.85**	-0.49	0.78	-0.78*	-0.71*	1.00		
Soil total nitrogen (STN)	-0.78*	-0.47	0.55	-0.63	-0.58	0.87**	1.00	
Soil pH	-0.41	-0.58	0.14	-0.56	-0.56	0.12	0.19	1.00

[†]*, $P < 0.05$;**, $P < 0.01$.

Table S2: The relationship between leaf caloric value (LCV) and mean annual temperture (MAT) and mean annual precipitaiton (MAP) at level of plant phylogenic family.

Serial number	Family	Correlation coefficient (R^2)	
		MAT	MAP
1[†]	*Labiatae*	0.07	0
2	*Compositae*	0.21	0.09
3	*Salicaceae*	0.06	0.03
4	*Liliaceae*	0.40	0.34
5	*Betulaceae*	0.17	0.09
6	*Umbelliferae*	0.01	0.11
7	*Pinaceae*	0.08	0.17
8	*Berberidaceae*	0.55	0.17
9	*Gramineae*	0.01	0.01
10	*Dryopteridaceae*	0.09	0.06
11	*Urticaceae*	0.08	0.21
12	*Euphorbiaceae*	0.54	0.64
13	*Ericaceae*	0.42	0.52
14	*Rutaceae*	0.88	0.66
15	*Theaceae*	0.99**	0.05
16	*Fagaceae*	0.51*	0.44
17	*Juglandaceae*	0.66**	0.78**
18	*Rosaceae*	0.84**	0.86**
19	*Sapindaceae*	0.72**	0.65*
20	*Ribesiaceae*	0.66*	0.76*
21	*Leguminosae*	0.56*	0.74**
22	*Malvaceae*	0.71*	0.69*

[†]The serial number of plant phylogenic families were derived from Fig.S5

[‡]*, $p < 0.05$; **, $p < 0.01$.

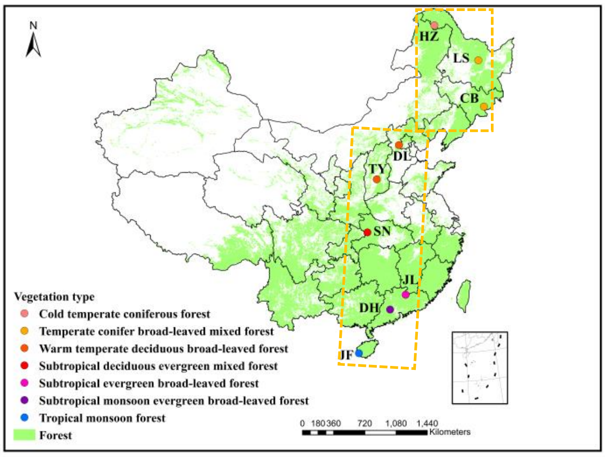

Figure S1: Geographic locations of the selected forests in this study. HZ, Huzhong; LS,Liangshui; CB, Changbai; DL, Dongling; TY, Taiyue; SN, Shennongjia; JL, Jiulian;DH, Dinghu; JF,Jianfengling. The figure was created by G.Y. Song using ArcGIS 9.2 (ESRI, USA; http://support.esri.com/Products/Desktop/arcgis-desktop/arcmap/10-4-1#downloads).

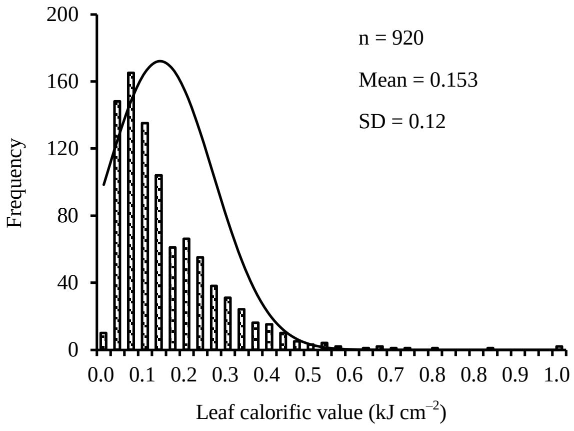

Figure S2: The frequency distribution histogram of leaf caloric value.

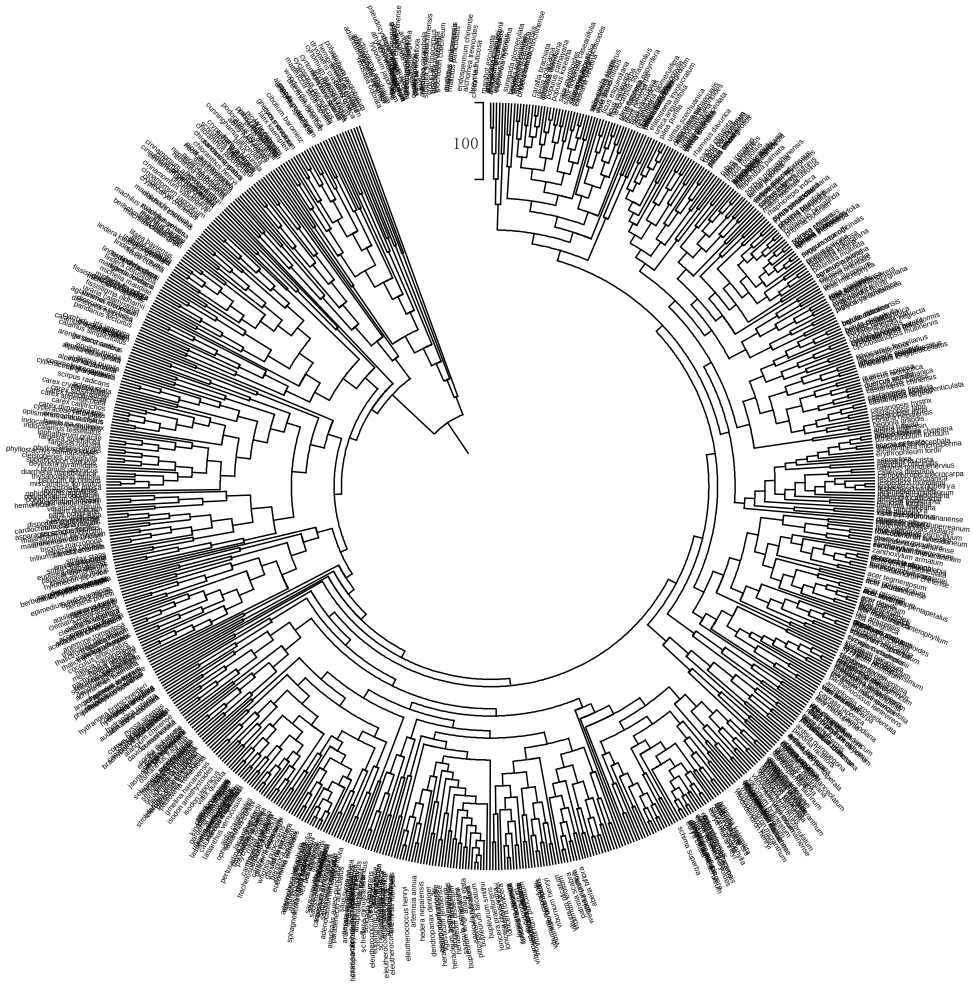

Figure S3: Phylogenetic tree of leaf caloric value from 745 plant species.

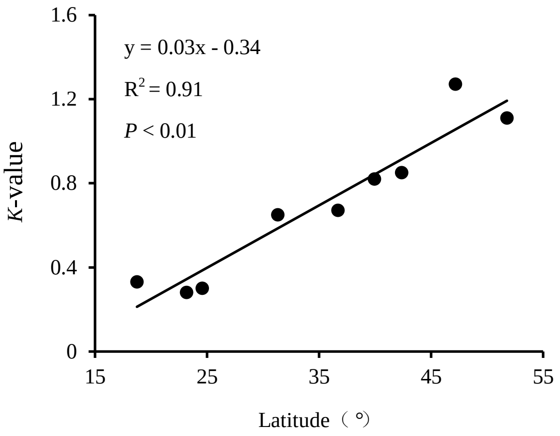

Figure S4: Variation in phylogenetic signal (*K*-value) along latitudinal gradients.

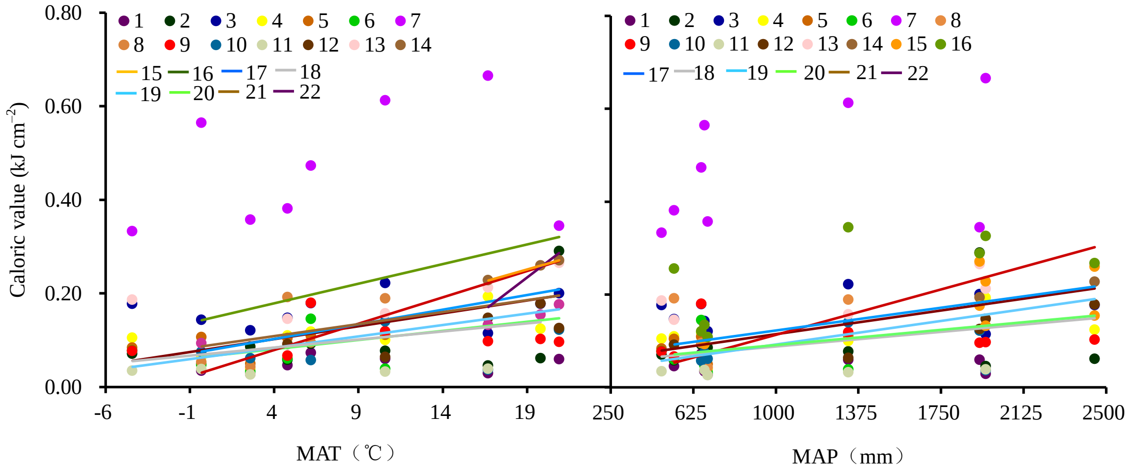

Figure S5: Changes in leaf caloric value with temperature and precipitation at family level. The family at least appeared at three forest types. The serial numbers of plant phylogenic family seeing Table S2; MAT, mean annual temperature; MAP, mean annual precipitation.

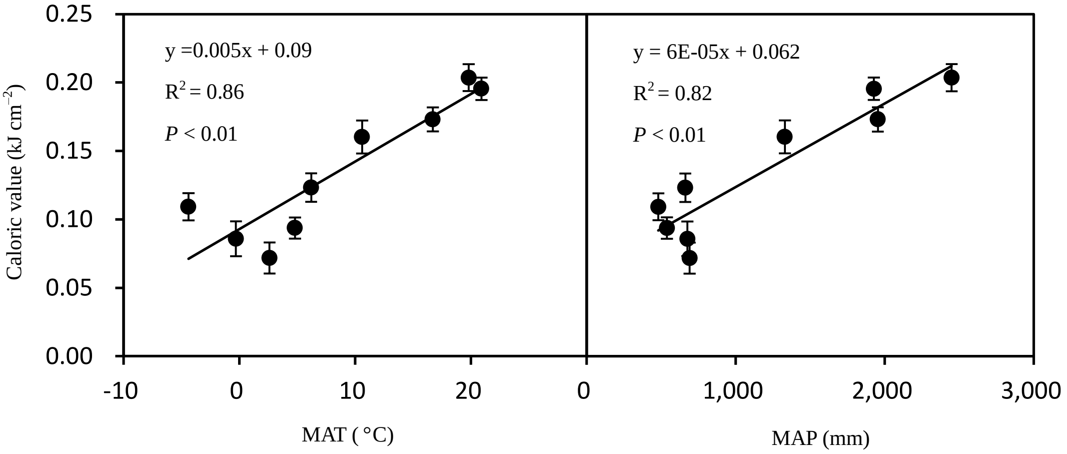

Figure S6: Changes in leaf caloric value with temperature and precipitation at nine plots (mean ± SE). MAT, mean annual temperature; MAP, mean annual precipitation.

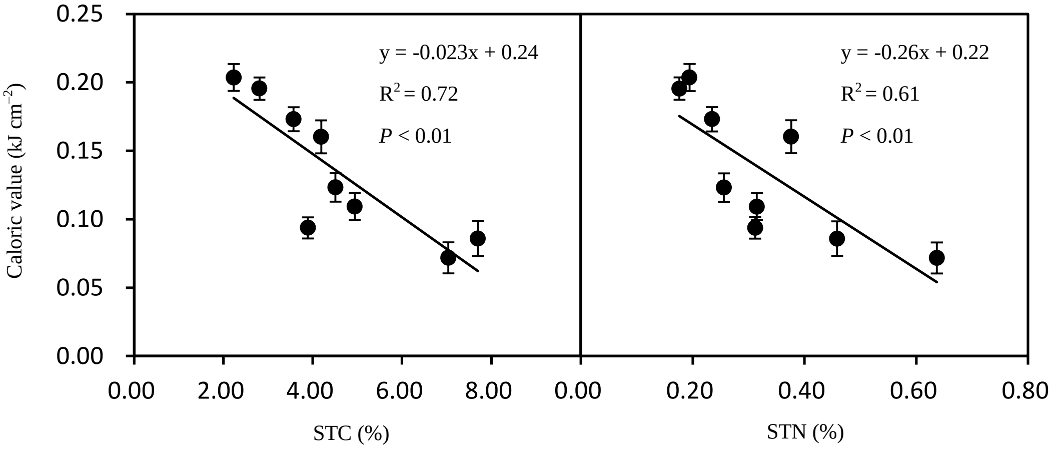

Figure S7: Changes in leaf caloric value with soil element content at nine plots (mean ± SE). STC, soil total carbon content; STN, soil total nitrogen content.
